# Supplementary material for: Preliminary Report of Nationwide COVID-19 Vaccine Compensation in Taiwan
Source: Healthcare (Basel). 2024 Jun 24;12(13):1250. doi: 10.3390/healthcare12131250 (PMC11241583; doi:10.3390/healthcare12131250)
Supplement: Supplementary file 1 [file healthcare-12-01250-s001.zip › healthcare-3005862-supplementary.pdf]

Supplemental Material

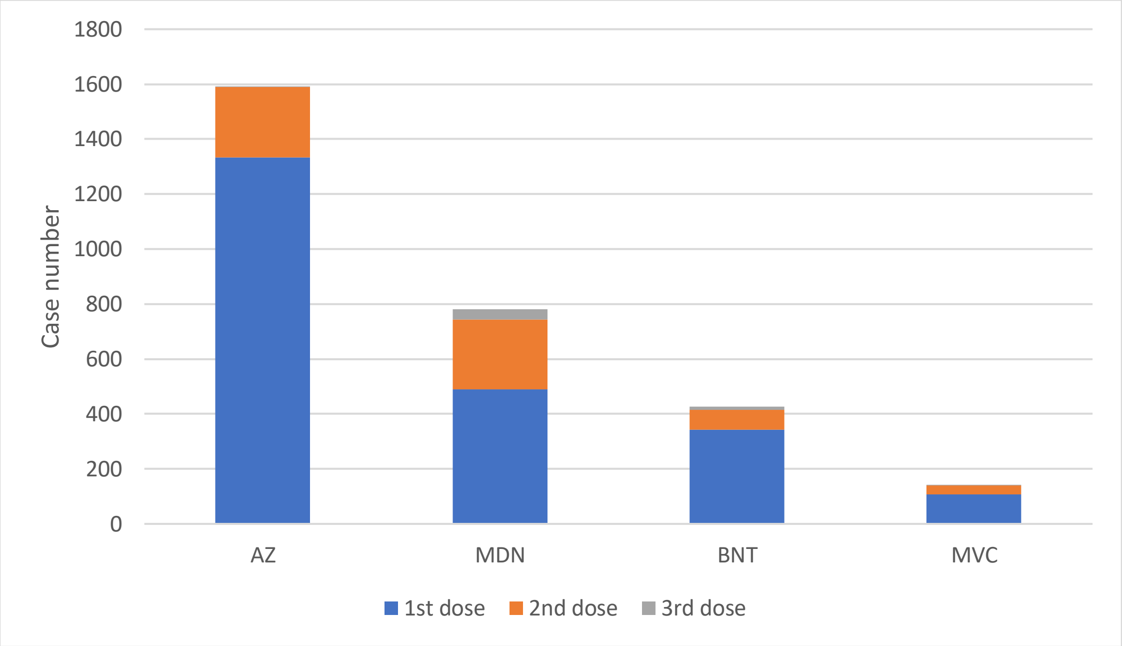

Figure S1. Types and doses of COVID-19 vaccines applied for compensation. Abbreviations: Oxford/AstraZeneca (AZ), Moderna (MDN), Pfizer/BioNTech (BNT) Medigen (MVC).

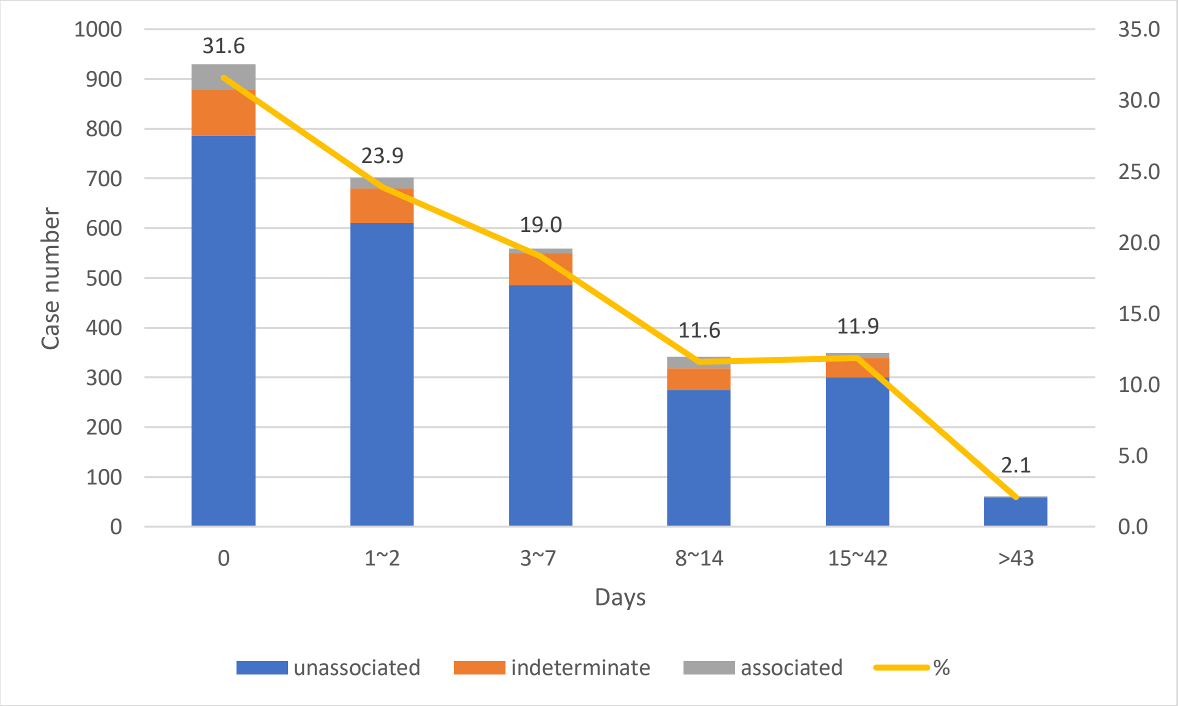

Figure S2. Time interval from vaccine administration to symptom onset and their association with the vaccination

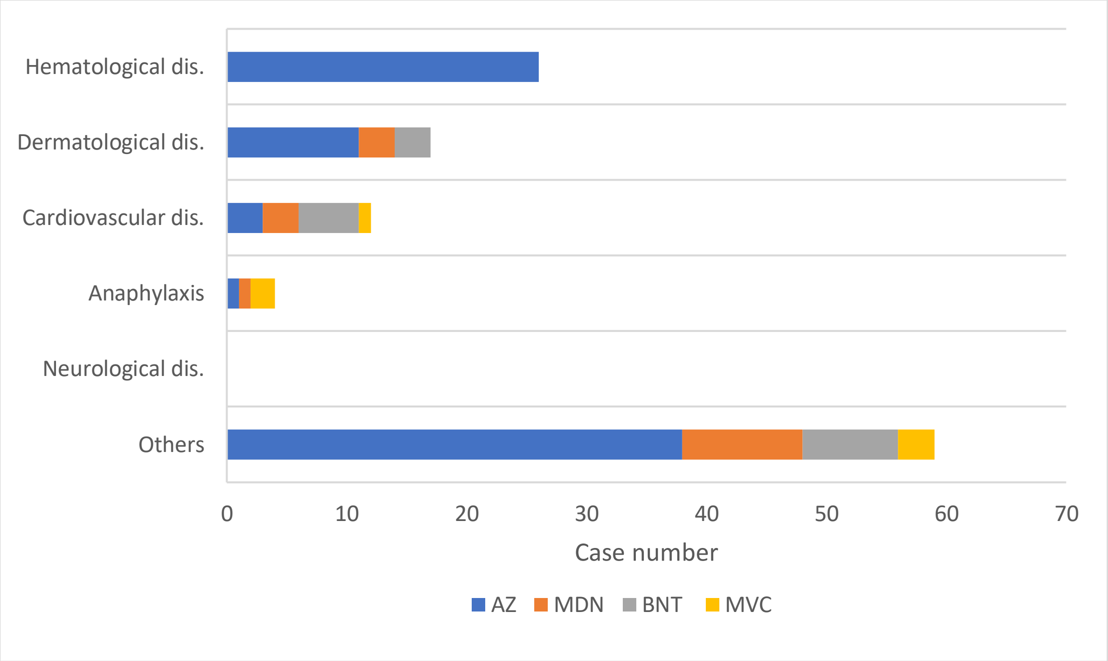

Figure S3. Diagnoses of problems and vaccine types in associated cases. Abbreviations: Oxford/AstraZeneca (AZ), Moderna (MDN), Pfizer/BioNTech (BNT) Medigen (MVC).

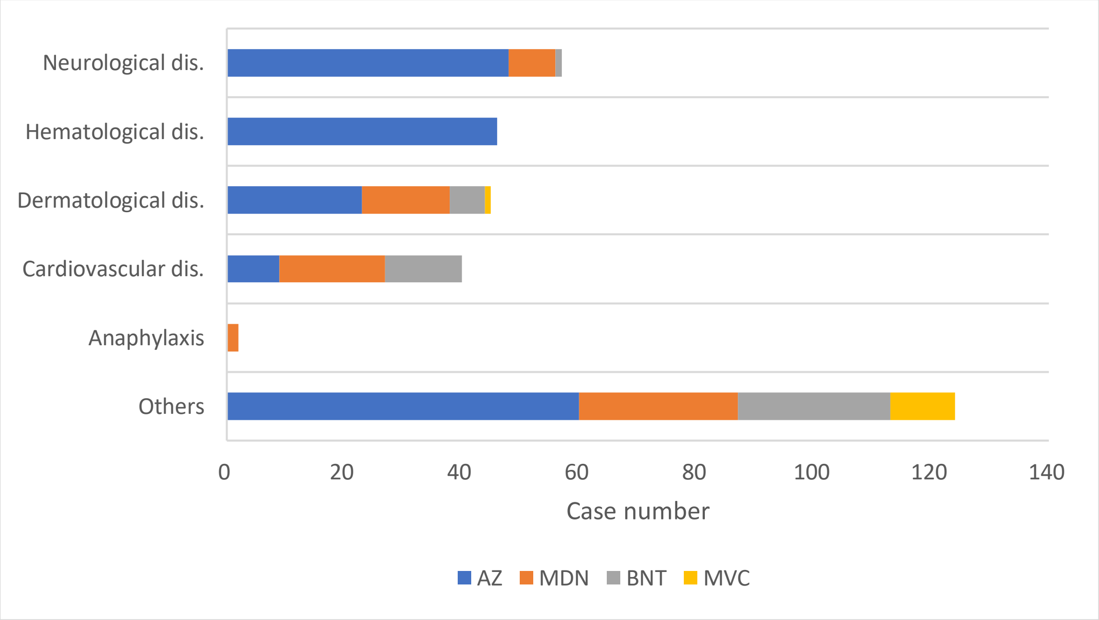

Figure S4. Diagnoses of problems and vaccine types in indeterminate cases. Abbreviations: Oxford/AstraZeneca (AZ), Moderna (MDN), Pfizer/BioNTech (BNT) Medigen (MVC).

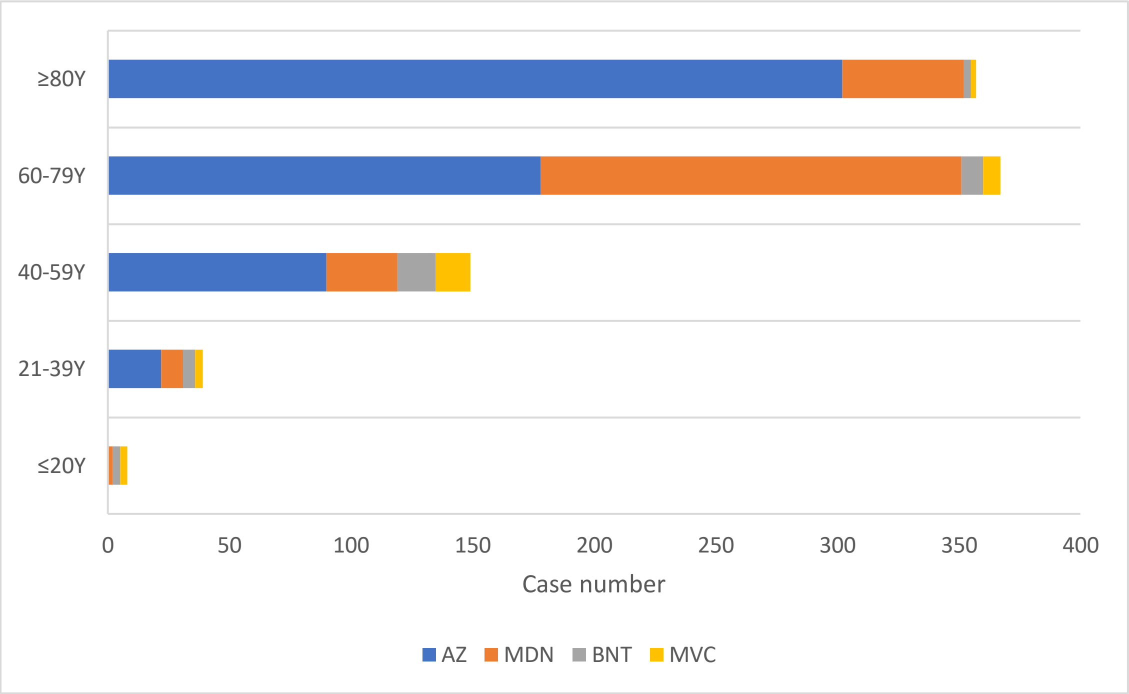

Figure S5. Age distribution of fatal cases. Abbreviations: Oxford/AstraZeneca (AZ), Moderna (MDN), Pfizer/BioNTech (BNT) Medigen (MVC).

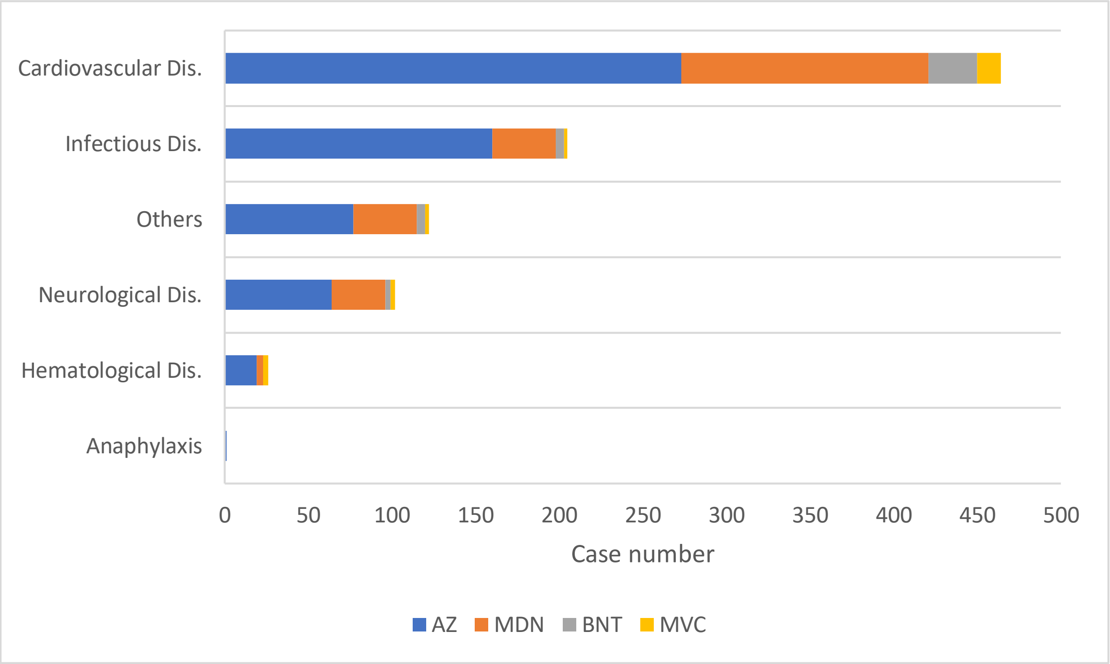

Figure S6. Diagnoses of problems in fatal cases. Abbreviations: Oxford/AstraZeneca (AZ), Moderna (MDN), Pfizer/BioNTech (BNT) Medigen (MVC).
